# Impact of mild traumatic brain injury (mTBI) on sperm genome integrity: insights from a mouse model

**DOI:** 10.1007/s40618-025-02549-w

**Published:** 2025-03-10

**Authors:** M. Memis, S. Taheri, Z. Y. Sukranlı, E. M. Duman, B. Er, Z. Hamurcu, Ahsen Güler, M. Rassoulzadegan, Z. Karaca, F. Tanriverdi, K. Unluhizarci, F. Kelestimur

**Affiliations:** 1 Betul-Ziya Eren Genome and Stem Cell (GENKOK) Center, Erciyes University, Kayseri, Türkiye; 2 Department of Medical Biology, Erciyes University Medical School, Kayseri, Türkiye; 3 Department of Cancer and Genetics, Cardiff University, Cardiff, UK; 4 Department of Endocrinology, Erciyes University Medical School, Kayseri, Türkiye; 5 Department of Endocrinology, Yeditepe University Medical School, Kadikoy, 34718 Istanbul, Türkiye

**Keywords:** Mild Traumatic Brain Injury (mTBI), Chromosome stability, Sperm, Telomeres, TERRA

## Abstract

**Purpose:**

Traumatic Brain Injury (TBI) poses a significant global health burden, with Mild TBI (mTBI) being the most prevalent form. TBI triggers activation of the hypothalamic–pituitary–adrenal (HPA) axis, which in turn affects the hypothalamic-pituitary–gonadal (HPG) axis regulating oogenesis and spermatogenesis. In this study, we investigated the impact of mTBI on sperm genome integrity using a repetitive mTBI (r-mTBI) mouse model.

**Methods:**

We assessed sperm telomere length (TL), free TERRA (fTERRA), and DNA/RNA hybrid TERRA (hTERRA) levels, alongside transcriptional changes in genes involved in TERRA regulation and DNA damage response.

**Results:**

Our findings reveal that a single mTBI event leads to a significant reduction in sperm TL during the acute phase, followed by an increase in TL during the chronic phase of r-mTBI, reminiscent of aging-associated changes. Moreover, we observed alterations in the transcription levels of *Rad51, Exo1, Rb1, RNaseH1*, and *RNaseH2* genes, particularly in association with fTERRA and hTERRA levels, following mTBI.

**Conclusion:**

Understanding the potential non-Mendelian effects of TBI holds promise for elucidating TBI pathogenesis, mechanisms of TBI-induced diseases, and conditions of unknown etiology. Given the risks associated with repeated TBI exposure, especially in sports like football and boxing, consideration of potential paternal transmission of effects to offspring is crucial.

**Supplementary Information:**

The online version contains supplementary material available at 10.1007/s40618-025-02549-w.

## Introduction

The primary function of a spermatozoa is to transfer the paternal hereditary function (Mendelian and non-Mendelian) to the embryo. Growing evidence from epidemiological studies suggests that environmental exposures and a father's lifestyle can affect the sperm and, subsequently, embryonic development, and health of offspring [1]. In mammals, exposure to certain environmental stressors or other factors can lead to impaired sperm and egg memory, which can have cascading effects over multiple generations [2, 3].

Gapp et al. showed that early traumatic stress causes long-term changes in special sets of miRNA and piRNA in spermatozoa, potentially influencing behavioural, metabolic and stress response variations in offspring through stress-induced glucose release [4]. Furthermore, their study revealed that when total RNA from spermatozoa isolated from the father was microinjected into fertilized oocytes resulted in a replication of the observed phenotypic changes in the offspring. Similarly, Christopher Morgan's team showed that chronic exposure to stress in adolescents and adults modifies the RNA profile of the spermatozoa and that nine of the miRNAs identified reduce the expression of certain target mRNAs in the zygote. These alterations induce a diminished activation of the Hypothalamus–Pituitary–Adrenal (HPA) axis in the adulthood [5].

The severity of traumatic brain injury (TBI) is assessed using the Glasgow Coma Scale and is classified as mild, moderate, or severe. Mild TBI (mTBI) is the most common type and can be difficult to diagnose and often unnoticed [6]. Today, mTBIs are common among adolescents and athletes participating in active sports, such as football, boxing, and kickboxing [7]. Recurrent mTBI in the athletes can lead to post-traumatic stress disorder, dementia, Alzheimer's disease, and pituitary deficiency [8, 9].

Activation of the HPA axis and persistent neuroinflammation resulting from TBI are recognized as catalysts for cellular aging [10, 11]. Moreover, aside from chronic stress, other environmental factors such as endocrine disruptors, or diet can alter the composition of small non-coding RNAs in spermatozoa, thereby impacting sperm development [3, 12].

The systemic effects of mTBI, although it is directly caused by a blow to the head [13] and disruption of hypothalamo-pituitary–gonadal (HPG) axis due to pituitary damage and/or hyperprolactinemia, might affect the process of spermatogenesis and oogenesis. However, it is not yet clear to what extent TBI affects the gamete genome and whether this effect can be passed on to the next generation.

Telomeres are the areas containing repeated motifs (TTAGGG^n^), its transcribed repeat RNA (CCCUAA^n^) and its protein complex (shelterin) located at the ends of chromosomes. The shortening process ends when telomeres approach the Hayflick limit, leading to cellular aging in most mammalian cells. Recent studies, however, affirm that telomere regulation can occur without the involvement of the telomerase enzyme [11, 14]. Studies have also shown that telomere length (TL) is transmitted from the father to the embryo during the first division stages, but that TL can be maintained by telomerase activity as early as the blastocyst stage [15].

Long non-coding RNAs (lncRNAs) known as telomeric repeat-containing RNA (TERRA) are transcribed from subtelomeric regions located at the ends of chromosomes. While telomeres were traditionally believed to be transcriptionally silent, recent research has unveiled the crucial role of TERRAs in genome stabilization and protection of chromosome ends from degeneration [16]. TERRA facilitates homologous recombination between telomeres at the ends of chromosome, and delays cellular senescence [17]. After being transcribed from the subtelomeric regions, a fraction of TERRA remains hybridized with the DNA at the telomeres from which it originates and regulate the activity of telomerase and therefore the length of the telomere, and the associated heterochromatinization, thus ensuring genome stability. Therefore, assessing TERRA expression necessitates quantification of both free and hybridized TERRA bound to DNA. Variations in TERRA expression levels are associated with altered TL, genomic instability, uncontrolled cell division, and cellular aging.

Harleen Hehar's group in 2017, examined whether TBI influences sperm telomeres [1]. They showed that age and paternal diet affect the phenotype of the offspring and mediate individual differences after TBI by modifying the DNA methylation of specific genes. They found no difference in sperm TL between the groups. They found that a single TBI changed the promoter methylation profile of the *Bdnf, Lept-R, Oxy-R, Tert, Igf2,* and *Igf2-R* genes in the spermatozoa [1].

To investigate the impact of TBI, a common occurrence among adolescents, young adults, and athletes, on telomere function and genome regulation at the spermatozoa level, we established mouse models of mTBI and repetitive mTBI (r-mTBI). Here, we report the evolution of sperm TL during acute and chronic phases the alteration of expression levels of TERRA (free) and hybrid TERRA fractions. Additionally, we show changes in the expression levels of *Rad51, Exo1, Rb1, RNase H1,* and *RNase H2* transcripts involved in the regulation of TERRA and the DNA damage response.

## Methods

**Fig. 1:**
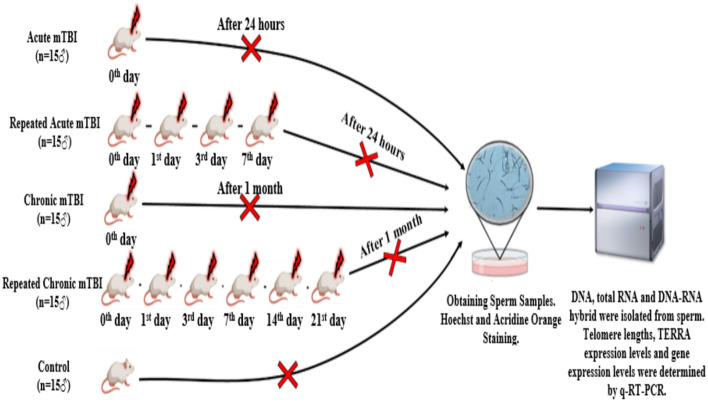
Experimental design

In this study, mTBI and r-mTBI mouse models were established in 2-month-old male *Balb/C* mice using the Marmaroau trauma model, and DNA damage analysis was performed by Hoechst and acridine orange staining in sperm cells of mice exposed to single mTBI (mTBI) and repetitive mTBI (r-mTBI) in the acute (day 1) and chronic (day 30) phases. In addition, TL was determined in sperm DNA samples of the groups; fTERRA and hTERRA expression levels were determined in total RNA and DNA-RNA hybrid samples; transcript levels of *Rad51, Exo1, Rb1*, *RNase H1*, and *RNase H2* genes involved in the regulation of TL and DNA damage response were determined in total RNA samples (Fig. 1).

### Animals

This study was carried out at the Betül-Ziya Eren Genome and Stem Cell (GENKOK) Centre of Erciyes University with the approval of the local ethics committee for animal experiments of Erciyes University (Decision No: 21/18). Male *Balb/C* mice bred in this center were used at two months with an average body weight of 30–40 g. The animals used in this project were maintained at a temperature of 20 ± 2 °C, a humidity of 52 ± 3%, 12 h light/dark cycle, fed with standard mouse pellet chow, and given free access to water by changing their bedding weekly.

### TBI mouse model

Trauma was induced in the mice according to the Marmarou trauma model, in which the mice were first given general anesthesia with a combination of 150 µl of 2% Rompun and 150 µl of Ketalar mixed in 2 ml of 0.9% NaCI. Then, a plastic disk was placed on the head. A 30-g weight was dropped from a height of 80 cm onto the skull [18]. While creating a r-mTBI model, mTBI was applied by shooting four times in total on the 0, 1, 3, and 7 days, including the first head trauma, for both the acute and chronic groups [19, 20]. After the trauma, the opened scalp of the mouse was sutured with 26 mm and 75 mm sutures (Surgisorb, England), and the mice were placed back in their cages. After the trauma model was created, vital functions were followed, and the r-mTBI acute group and m-TBI acute groups were sacrificed 24 h after the last application, while the r-mTBI chronic and mTBI chronic groups were sacrificed 30 days after the last application. For the control group, it was sacrificed without any application to mice. Following establishing the acute and chronic phase mTBI mouse model (Fig. 1), the mice were sacrificed painlessly using cervical dislocation [21], and sperm samples were collected from the vas deferens.

### Hoechst staining

Sperm samples were taken into a 15 ml falcon tube. The sperm samples in the falcon tube were mixed for 5 min. The falcon tube was centrifuged at 1000 rpm for 2 min at + 4 °C, and the supernatant was taken into a new falcon. 10 µl of the sperm sample in the falcon was taken, and sperm count was performed on a Thoma slide. The sperm count was taken into 1.5 ml Eppendorf with 800.000–1.000.000 sperm per milliliter. The sperm in the new tube was centrifuged at 4000 rpm for 5 min at + 24 °C. After centrifugation, the supernatant was discarded, and 500 µl of 4% paraformaldehyde was added. It was fixed for one hour at room temperature in the dark. After fixation, the supernatant was centrifuged at 4000 rpm for 5 min at + 24 °C. After centrifugation, the supernatant was discarded, and sperm samples were washed by adding 500 µl PBS. This process was repeated twice. After washing, the supernatant was discarded. The sperm samples were resuspended by adding 100 µl PBS to the pellet. The resuspended sperm samples were spread on a slide and allowed to dry at room temperature for 30 min. The dried sperm samples were stained with 200 µl of bisbenzimide (Sigma, Cat. No.861405) for each slide and kept in the dark for 5 min. The slide was washed with PBS, and images were taken on a Fluorescence Microscope Nikon Ti Eclipse (Nikon, Japan) 40 × objective. After the pictures were taken, the number of damaged spermatozoa was determined and analyzed using the ImageJ program [22, 23].

### Acridine orange staining

The sperm count was taken into a 1.5 ml eppendorf with 800.000–1.000.000 sperm per milliliter. Sperm samples taken into a new tube were centrifuged at 4000 rpm for 5 min at + 24 °C. After centrifugation, the supernatant was discarded, and sperm samples were washed by adding 500 µl PBS. This process was repeated twice. After washing, the supernatant was removed. The sperm samples were resuspended by adding 100 µl PBS to the pellet. The resuspended sperm samples were spread on a slide and dried at room temperature for 20 min. The dried sperm samples were fixed by coating the slide surface with 200 µl Carnoys solution (3:1 ratio of methanol: glacial acetic acid) for each slide. The slides with sperm samples were allowed to dry for 2 h at room temperature. To stain the sperm samples, a staining solution was prepared using 10 ml of acridine orange, 40 ml of 0.1 M citric acid, and 2.5 ml of Na_2_HPO_4_−7H_2_O [24]. 200 µl of the staining solution was added to the dried slide, stained, and kept in the dark for 5 min. The slide was washed with PBS, and images were taken with a 40 × objective of a Fluorescence Microscope Nikon Ti Eclipse (Nikon, Japan). After the pictures were taken, the number of damaged spermatozoa was determined and analyzed using the ImageJ program [24].

### Total RNA isolation

Sperm samples were placed in 15 ml falcon tubes and mixed for 5 min. The samples were centrifuged at 1000 rpm for 2 min at 4 °C, and the supernatant was transferred to a new falcon tube. The supernatant was further centrifuged at 3000 rpm for 15 min at 4 °C, and the supernatant was discarded. 500 µl of dH_2_O and 4.5 ml PBS were added, and the samples were centrifuged at 3000 rpm for 15 min at 4 °C. The supernatant was discarded, and 3 µl of 0.1 M DTT (Dithiothreitol) and 1000 µl of Trizol were added to the pellet and kept at −20 °C for 5 min. The pellet solution was transferred to a 1.5 ml eppendorf. 200 µl of chloroform was added and centrifuged at 12,000*g* for 15 min at 4 °C. The upper aqueous phase was transferred to a new tube, and a 1:1 ratio of 2-propanol was added to the supernatant and kept at −20 °C for 30 min. Also, the interphase was transferred to a different tube for DNA isolation. The RNA samples were centrifuged at 12,000*g* for 10 min at 4 °C. The supernatant was discarded, 1 ml of 70% ethanol was added, and the samples were centrifuged at 7,500*g* for 5 min at 4 °C. The supernatant was discarded, and 30 µl of nuclease-free water was added to the pellet, which was then resuspended. The obtained RNA sample concentrations and purity were determined using a Shimadzu-Biotech Bio-Spec Nano Spectrophotometer. RNA samples, which have been determined concentrations, were stored at − 80 °C until the experiments begins [25–27].

### DNA isolation

In total RNA isolation, interphase was transferred to a different tube, and then a 1:1 ratio of 2-Propanol was added, and the DNA samples were kept at −20 °C for 30 min and centrifuged at + 4 °C, 12,000 g for 30 min. After centrifugation, the supernatant was discarded, and 1 ml of 70% ethanol was added and centrifuged at 7500*g* for 5 min at + 4 °C. After centrifugation, 600 µl Cell Lysis Buffer containing 1 M Tris, 14% SDS, 0.1 M EDTA, and 40 µl Proteinase K was added and incubated overnight at 56 °C. 100 µl of ammonium acetate was added and centrifuged at 4000 rpm for 20 min at + 18 °C. The aqueous phases were transferred to a new 1.5 ml eppendorf, and 2-Propanol was added to the supernatant at a ratio of 1:1. Then centrifuged at 12,000*g* for 10 min at + 4 °C. After centrifugation, the supernatant was discarded, and 1 ml 70% ethanol was added and centrifuged at 4000 rpm for 10 min at + 4 °C. After centrifugation, nuclease-free water was added, and the pellet was resuspended. DNA sample concentrations and purity were determined using Shimadzu-Biotech Bio-Spec Nano Spectrophotometer. DNA samples were stored at − 80 °C until the experiments started [25–27].

### DNA-RNA hybrid isolation from sperm DNA samples

To the DNA-RNA Hybrids isolated from sperm DNA samples, 10 µl DNase (Roche, Cat. No.4716728001, Germany) and 90 µl DNase incubation buffer was added to the DNA and left to incubate at room temperature for 15 min. After incubation, 300 µl Trizol and 180 µl chloroform were added and centrifuged at 12,000 *g* for 15 min at + 4 °C. The upper aqueous phase was taken into a new 1.5 ml eppendorf, and 2-Propanol was added to the supernatant at a ratio of 1:1. Then centrifuged at 12,000 g for 10 min at + 4 °C. After centrifugation, the supernatant was discarded, and 1 ml 70% ethanol was added and centrifuged at 7500*g* for 5 min at + 4 °C. After centrifugation, nuclease-free water was added to the isolated DNA-RNA hybrid tube. DNA-RNA hybrid sample concentrations and purity were determined using Shimadzu-Biotech Bio-Spec Nano Spectrophotometer. DNA-RNA hybrid samples were stored at − 80 °C until the experiments started [25–27].

### Determination of telomere length (TL)

Telomere length (TL) was determined using the DNA obtained from sperm samples using the telomere standard, 36B4 standard, telomere primers, and 36B4 primers (presented in Supplementary Table 1) using the Real-Time qPCR method. Stock telomere and 36B4 standards were diluted for each reaction, as in Supplementary Table 2, to form a standard series. After the standards were prepared, 1 µl of pGL3-Basic Vector (Promega, E1751, France) was added to all standards. After the standards were prepared, separate reaction mixes were prepared for telomeres and 36B4 [28].

The reaction mix was prepared according to the manufacturer’s instructions: for the reaction mix, 10 µL of SYBR Green mix (2X), 5 µL of nuclease-free water, and 1 µL of 10 pmol primers were mixed. After preparing the reaction mix, 16 µl of the mix was distributed to the 96-well plate for each sample, and 4 µl of DNA sample was added. The reaction was then performed in Roche LightCyler LC480 ll (Germany) under the PCR conditions presented in Supplementary Table 3.

TL were calculated using the Ct values obtained for the telomere standard and 36B4 standard as a result of PCR, and the standard curve graph was drawn using the method of Callagan et al. [29].

### Determination of TERRA expression levels in total RNA and hybrid DNA-RNA and determination of mRNA expression levels by real-time qPCR

To determine the expression levels of free TERRA (fTERRA), hybrid TERRA (hTERRA) (from DNA-RNA hybrids), *Rad51, Exo1, Rb1*, *RNase H1*, and *RNase H2,* cDNA synthesis was performed according to the manufacturer's protocol (Roche, 07912374001, Germany). Briefly, 4 µL of Reaction Buffer (5X), 10 µL of nuclease-free water, and 5 µL of RNA, which concentrations were adjusted to be between 10 ng-100 ng. The prepared mix was vortexed very briefly. Then 15 µl of the mix was dispensed into each well, and 5 µl of total RNA sample was added (hTERRA levels were determined from DNA-RNA hybrid samples isolated from DNA samples). After this process, 2 µl of Enzyme mix (Reverse Transcriptase) was added to the products. Then they were placed in a thermal cycler at 42 °C 15 min 85 °C 5 min 65 °C 15 min program [28].

Expression levels in cDNA samples were determined by using the SYBR Green I Master kit (Roche, 04707516001, Germany) according to the manufacturer's protocol. Briefly, 10 µL of SYBR Green mix (2X), 5 µL of nuclease-free water, and 1 µL of 10 pmol primers (presented in Supplementary Table 4) were mixed and dispensed into 96 multi-well plates, and 4 µl of cDNA sample was added. The reaction was then performed in Roche LightCyler LC480 ll (Germany) under the PCR conditions presented in Supplementary Table 3. All samples were run in duplicate to rule out errors due to manipulation. *Gapdh* was used as the housekeeping gene. Changes in gene expression were determined by using the 2^−ΔΔCt^ method [28, 30, 31]

### Determination of hormone levels by ELISA methods

Blood samples collected from mice were centrifuged at 3000 rpm for 20 min for phase separation, and serum samples from the upper phase were transferred to the new reaction tubes and stored at −80 °C until the assay. Serum hormone levels were measured by using the ELISA kits for GH (Sunred, Shanghai Cat No: 201020677), ACTH (Ylbiont, Shanghai, Cat No: YLA1790MO), IGF-1(Sunred, Shanghai, Cat No:201020038), CORT (Ylbiont, Shanghai, Cat No: YLA0342MO) and DHEA-S (Ylbiont, Shanghai, Cat No: YLA0536MO) ELISA kits. Serum hormone levels were determined in accordance to our previous study and also the manufacturer's instructions [32].

### Statistical analysis

After the results were obtained, comparisons were made between the experimental and control groups. The suitability of the data for normal distribution was evaluated by the histogram, q-q graphs, and Shapiro–Wilk test. Depending on whether the data showed normal distribution or not, One Way ANOVA, Kruskal Walls, student t-test, Mann Whitney-U test, and correlation analyses with Pearson and Spearman tests were performed. Data were analyzed using SPSS version 22 (IBM, USA) and Graph-Pad Prism 8 software. Results with p values < 0.05 were considered statistically significant [28, 33].

## Results

### Hoechst and acridine orange staining reveal no major DNA damage in spermatozoa after TBI

**Fig. 2:**
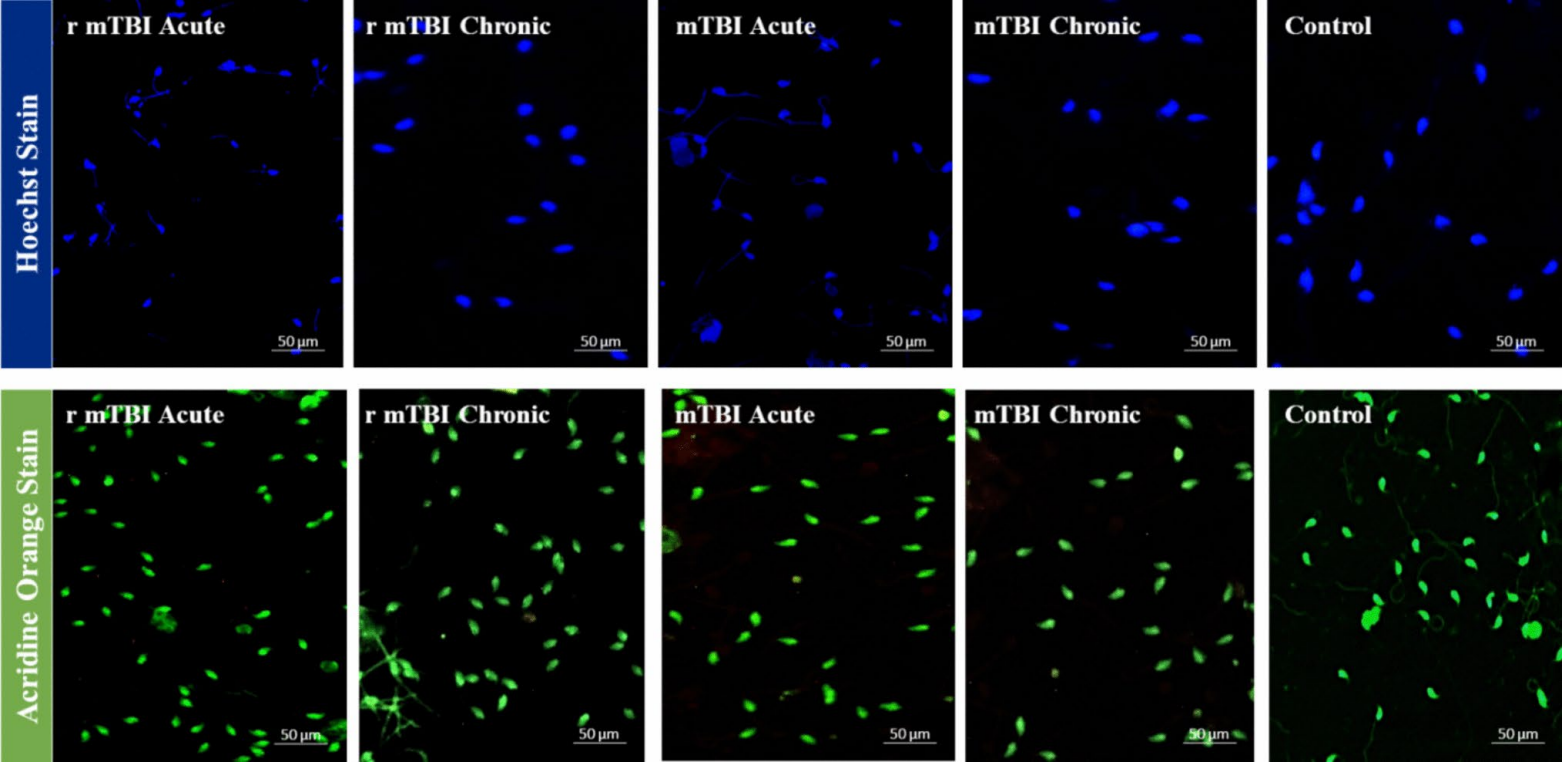
Hoechst and Acridine Orange staining results on spermatozoa from acute r-mTBI, chronic r-mTBI, mTBI-acute, mTBI-chronic,and control groups. (Images were captured from 10 distinct regions within each sample. A total of 300 sperm cells per sample were assessed across these images)

To assess the impact of mTBI on sperm DNA integrity, we conducted Hoechst and Acridine orange staining assays on sperm samples collected from five mice per experimental group, which included mTBI-acute, mTBI-chronic, r-mTBI-acute, r-mTBI-chronic, and control groups. Our objective was to determine the presence of significant DNA damage resulting from mTBI. While we did not observe notable DNA damage in sperm samples from the mTBI and r-mTBI groups, we did notice a reduction in Acridine orange staining intensity in chronic mTBI samples (Fig. 2).

### Telomere length (TL) in sperm DNA samples are affected by r-mTBI

**Fig. 3:**
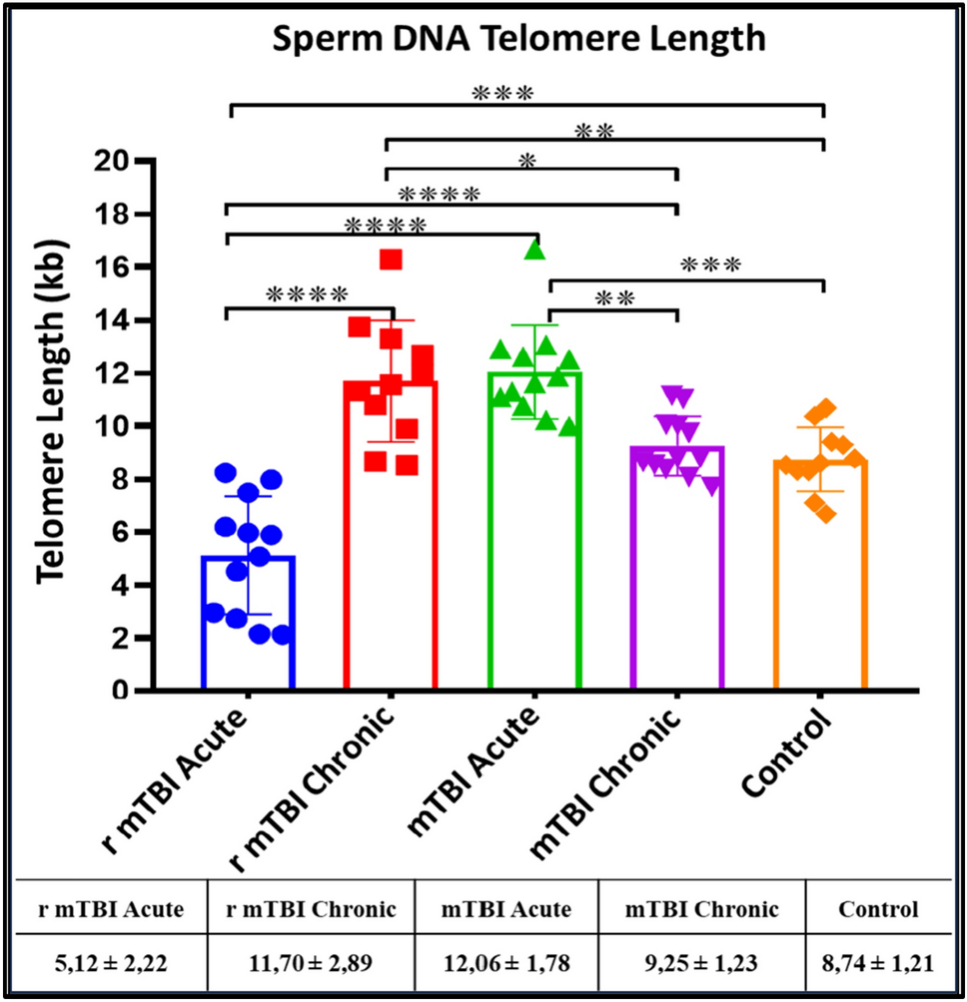
Comparison of TL in sperm samples from mTBI and r-mTBI groups (*p < 0.05, **p < 0.01 ***p < 0.001 ****p ≤ 0.001)

TL was determined in DNA samples isolated from spermatozoa from the acute and chronic mTBI and r-mTBI groups. It was found that the TL of the r-mTBI-acute group (p < 0.001) was shorter than that of the control group. The TL of r-mTBI-chronic (p ≤ 0.0001) and mTBI-acute (p < 0.001) groups were also found to be increased compared to the control group. The TL of the r-mTBI -chronic group was found to be increased compared to the control group (Fig. 3).

### Expression levels of TERRA (fTERRA) free are altered after TBI

**Fig. 4:**
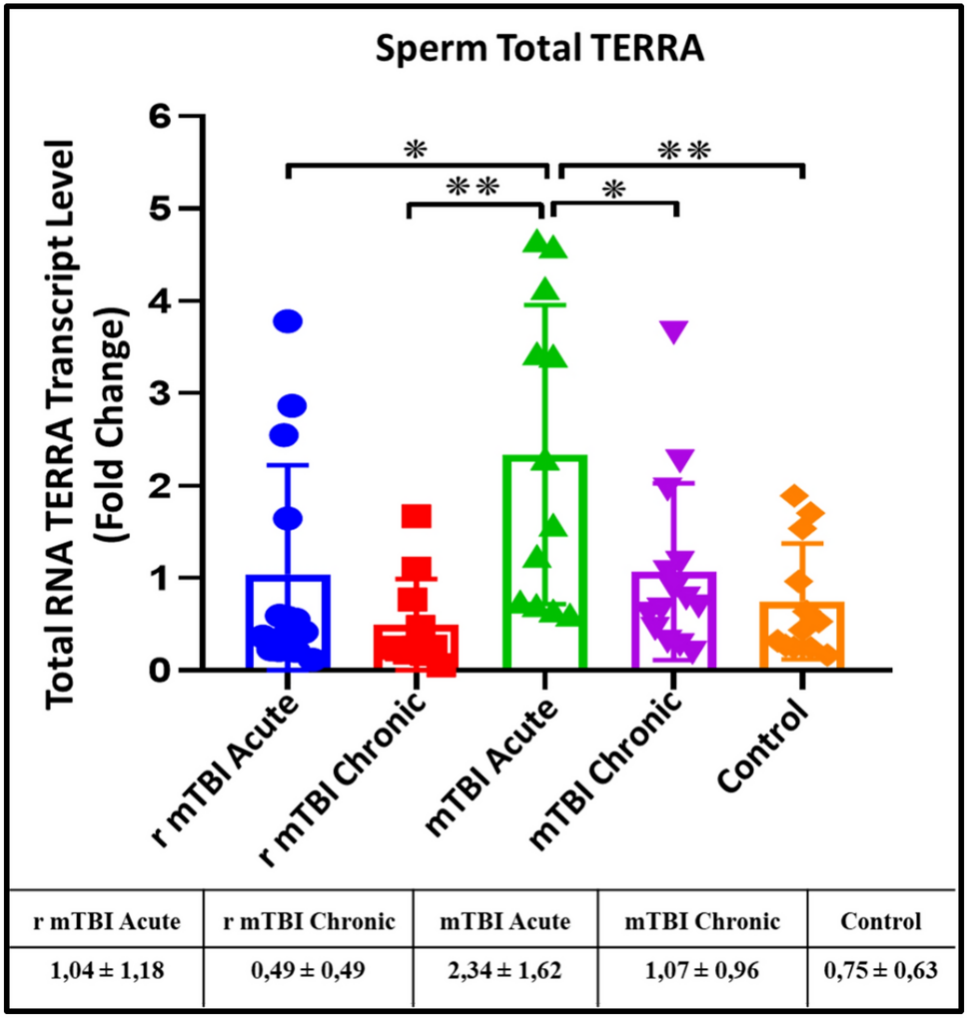
Comparison of fTERRA xpression levels in total RNA samples isolated from spermatozoa of acute and chronic groups after mTBI and r-mTBI (*p < 0.05, **p < 0.01)

When fTERRA levels were determined from TRizol-extracted RNA samples, it was found that fTERRA expression levels increased significantly in the mTBI-acute group compared to r-mTBI-acute (p ≤ 0.05), r-mTBI-chronic (p ≤ 0.01), mTBI-chronic (p ≤ 0.05) and control (p ≤ 0.01) groups (Fig. 4).

### DNA-RNA Hybrids, (hTERRA) expression levels are only affected after chronic TBI

**Fig. 5:**
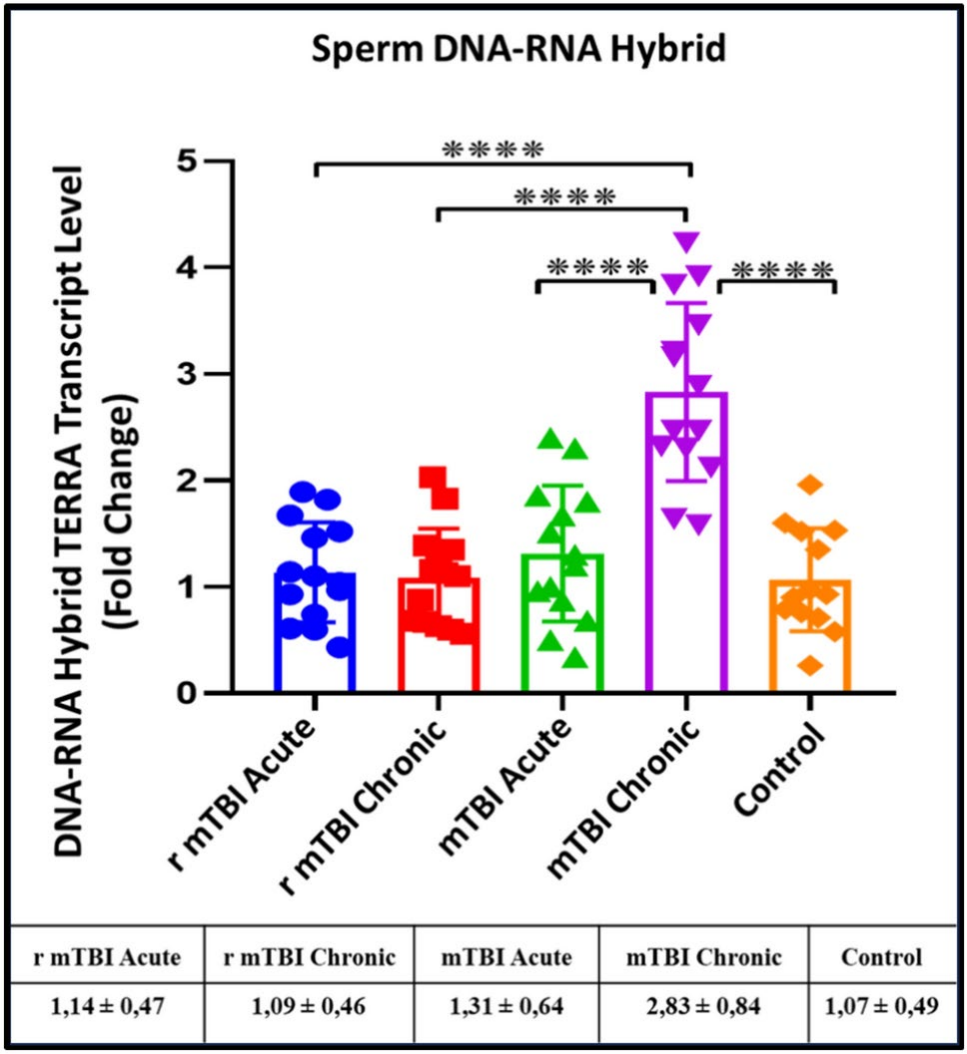
Expression levels of hTERRA in DNA-RNA hybrid samples isolated from sperm DNA samples of acute and chronic groups after mTBI and r-mTBI (****p ≤ 0.0001)

When hTERRA expression levels in DNA-RNA hybrid samples are compared between groups, hTERRA levels are not much affected in the acute phase, whereas they respond by increasing in the chronic phase of mTBI. However, in the chronic r-mTBI group, the levels harmonize and return to normal. These results rise the question what exactly is going on behind the scenes? (p ≤ 0.0001) (Fig. 5).

### Transcript levels of *Rad51, Exo1, Rb1, RNase H1,* and *RNase H2* genes in sperm samples from acute and chronic groups are altered after mTBI and r-mTBI

**Fig. 6:**
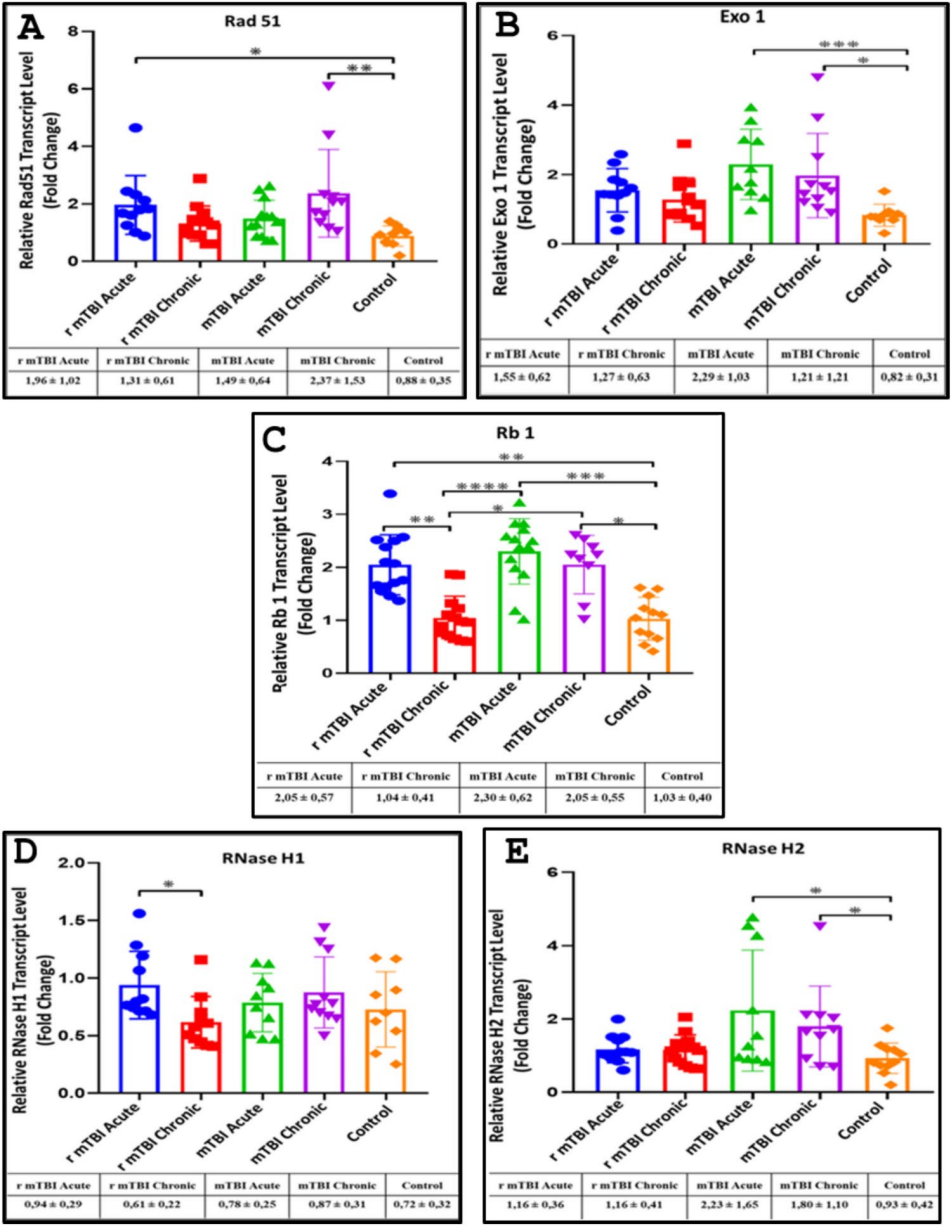
Transcript levels of *Rad51, Exo1, Rb1, RNaseH1*, and *RNaseH2* genes in sperm samples from acute and chronic groups after mTBI and r-mTBI. **A***Rad51* gene transcript levels. **B***Exo1* gene transcript levels. **C***Rb1* gene transcript levels. **D***RNaseH1* gene transcript levels. **E***RNaseH2* gene transcript levels (*p < 0.05, **p < 0.01, ***p < 0.001, ****p ≤ 0.0001)

In our study, we investigated the transcript levels of several key genes involved in DNA repair pathways and telomere regulation across different groups, including acute and chronic mTBI and r-mTBI, compared to a control group. We found that *Rad51*, a crucial player in DNA double-strand break repair, exhibited increased transcript levels in all groups compared to the control, with statistically significant elevation observed in the chronic mTBI (p < 0.01) and acute r-mTBI (p < 0.05) groups (Fig. 6A). Notably, *Rad51* levels correlated positively with hybrid TERRA (hTERRA) in the chronic phase of mTBI.

Exonuclease 1 (Exo1), contributing to DNA repair pathways such as nucleotide excision repair, also displayed elevated transcript levels across all groups, with statistically significant increases noted in the acute mTBI (p < 0.001) and chronic mTBI (p < 0.05) groups (Fig. 6B). In the context of telomeric chromatin modification, the transcript levels of *Rb1* (Retinoblastoma transcriptional corepressor 1), a transcription factor involved in telomere regulation through TERRA expression, were found to be increased in all groups except chronic r-mTBI compared to the control (p < 0.01, p < 0.001, p < 0.05) (Fig. 6C).

Further analysis revealed the cooperative role of *Exo1* with hTERRA in the chronic phase of mTBI, while *Exo1* primarily mediated the response during the acute phase of mTBI. Additionally, RNaseH1, responsible for degrading RNA engaged in hybrids with DNA (R-loop), exhibited a statistically significant increase in transcript levels in the acute r-mTBI group compared to the acute mTBI group (p < 0.05) (Fig. 6D).

Regarding *RNaseH2*, which shares similar activity with *RNaseH1* and is implicated in regulatory pathways during mTBI, transcript levels were significantly elevated in both acute and chronic mTBI groups compared to the control (p < 0.05) (Fig. 6E). Notably, *RNaseH2* appeared to be unresponsive during repeated mTBI.

### Serum hormone levels associated with or secreted by the pituitary gland from acute and chronic groups after mTBI and r-mTBI

TBI is associated with various endocrine abnormalities, including pituitary dysfunction. These effects may affect not only the cells related to the endocrine system but also all body cells, including gamete cells, and can lead to cellular aging and changes in DNA stability. Understanding the prevalence of pituitary dysfunction and temporal patterns of these dysfunctions is crucial for effective clinical evaluation and management. In this study, we have measured the serum hormone levels of Adrenocorticotropic Hormone (ACTH), Cortisol (CORT), Dehydroepiandrosterone Sulfate (DHEA-S), Growth hormone (GH) and Insulin-like Growth Factor 1 (IGF-1) in mTBI and r-mTBI groups.

**Fig. 7:**
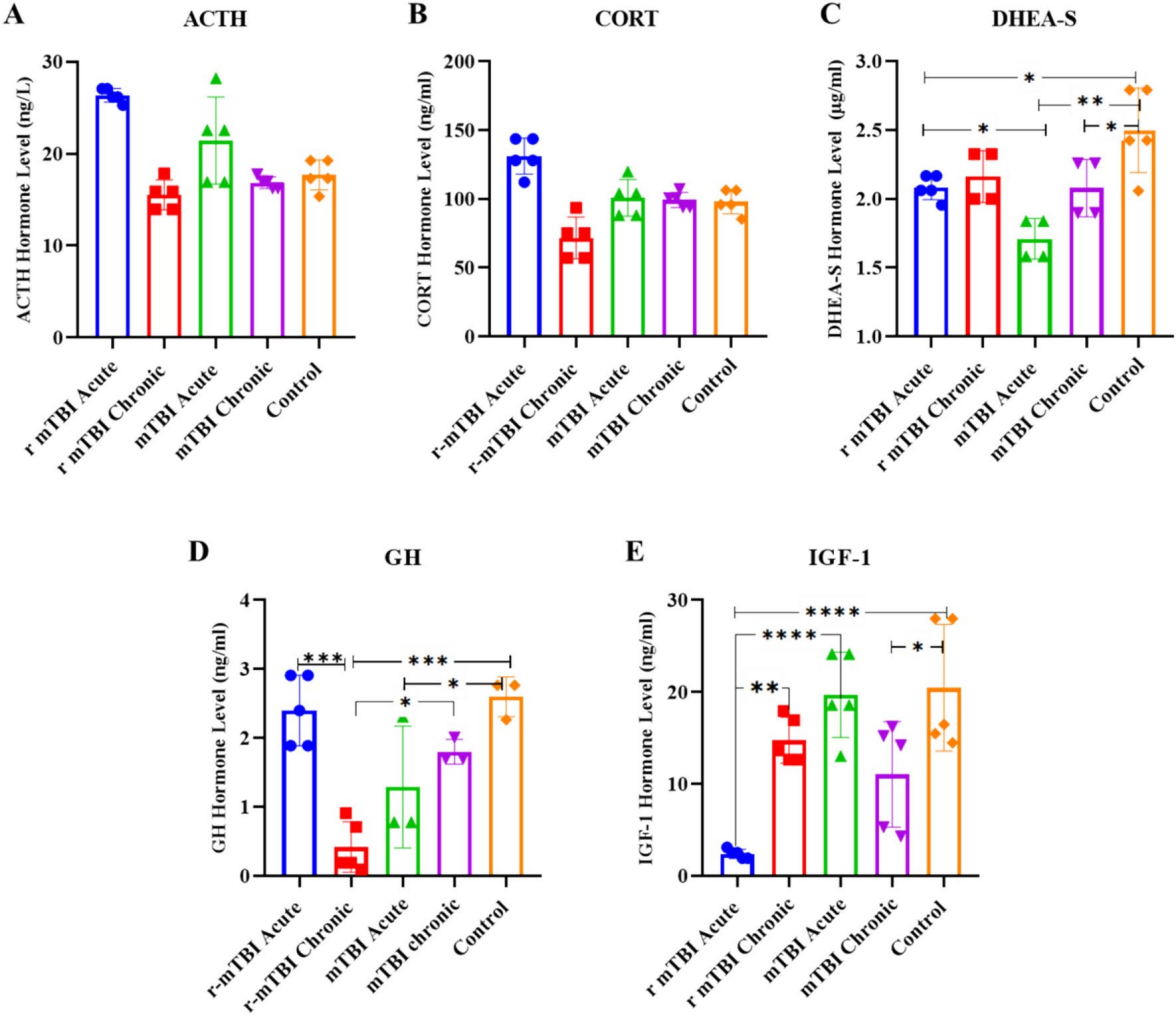
Evaluation of serum hormone levels associated with or secreted by the pituitary gland in the acute and chronic groups following mTBI and r-mTBI. **A** ACTH hormone levels. **B** CORT hormone levels. **C** DHEA-S hormone levels. **D** GH hormone levels. **E** IGF-1 hormone levels

In the mTBI and r-mTBI models, serum samples were collected during the acute and chronic phases, and the levels of, ACTH, CORT, DHEA-S, GH and IGF-1were analyzed. When the ACTH and CORT levels of the mTBI groups were compared with each other, no significant difference was detected between them (Fig. 7A, B).

In the comparison of DHEA-S levels across the groups, we found that DHEA-S levels in the r-mTBI acute, mTBI acute, and mTBI chronic groups were significantly reduced in comparison to the control group. Furthermore, DHEA-S levels in the mTBI acute group were found to be significantly lower than those in the r-mTBI acute group (Fig. 7C).

When GH hormone levels were compared between the groups, we found that GH levels decreased significantly in the r-mTBI chronic and mTBI acute groups compared to the control group, with the greatest decrease being in the r-mTBI chronic group (Fig. 7D).

Regarding IGF-1 levels across groups, we found that serum IGF-1 levels in the r-mTBI acute, mTBI chronic, and r-mTBI-chronic groups were significantly lower than those in the control group. The greatest decrease in IGF-1 levels was found in the r-mTBI acute group (Fig. 7E).

As a result of the correlation analysis, we found that there was a significant negative correlation between hTERRA levels and, ACTH, CORT, DHEA-S and IGF-1, levels in the mTBI acute group (Supplementary Table 5).

No significant correlation was found between TL, hTERRA, and fTERRA levels, and hormone levels in other groups (Supplementary Table 6, Supplementary Table 7, Supplementary Table 8).

## Discussion

This study created mild traumatic brain injury (mTBI) and repeated mTBI (r-mTBI) mouse models. Serum hormone levels related to the HPA and GH axes in the acute and chronic phases of mTBI and r-mTBI were studied to show that mTBI disrupts hormone regulation, and then the effects of mTBI, especially repeated mTBI, on sperm telomere length (TL), hybrid TERRA (hTERRA), and free TERRA (fTERRA) levels in the acute and chronic phases were investigated. As a result of the study, the change in sperm telomere regulation and the changing hormone levels indicate that mTBI may affect gamete cells and the next generation through gamete cells. These changes are implicated in TL regulation, essential for genome stability maintenance [34]. On the other hand, lack of serum FSH, LH, testosterone levels due to limited amount of serum samples are the limitation of the study.

mTBI is associated with various endocrine abnormalities, including possible pituitary dysfunction [35]. These effects may affect not only cells associated with the endocrine system but also all body cells, including gamete cells, and may lead to changes in genome stability. Understanding the prevalence and temporal patterns of these dysfunctions is crucial for effective clinical management. In this study, firstly, serum hormone levels related to the HPA and GH-IGF-1 axes were evaluated. After mTBI, there was no significant difference between the groups in terms of ACTH and CORT levels, while DHEAS hormone levels were shown to decrease in the acute and chronic phases after both acute and repeated mTBI, and GH levels were shown to decrease after acute mTBI, especially after repeated mTBI. IGF-1 hormone levels were shown to decrease in both acute and chronic phases after both single mTBI and repeated mTBI.

While we observed an increase in TL following mTBI within the acute mTBI group, this elongation reverted to control levels in the chronic mTBI group. Interestingly, in the chronic r-mTBI group, the heightened TL was sustained. Conversely, the acute repetitive mTBI group exhibited the shortest telomeres. It's worth noting that telomeres tend to be longer in older individuals compared to younger ones [36]. This raises questions about whether the increased TL in sperm could be indicative of premature aging. However, the implications of this phenomenon on subsequent generations and its regulation post-zygote formation remain unclear.

Our investigation unveiled marked elevations in TRIzol-extracted TERRA (free) transcripts within mouse spermatozoa post-acute mTBI, a novel observation in the literature. This discovery underscores the potential significance of TERRA transcripts in the initial response to TBI, possibly impacting telomere elongation dynamics.

After the elucidation of DNA's classical double helix structure, speculation arose regarding the existence of alternative stable structures, including a triple helix comprising single-stranded DNA or RNA. These triplex structures, formed by inserting a third strand into the major groove and establishing additional hydrogen bonds with DNA base pairs (known as Hoogsteen base pairs), were eventually discovered both in vitro and in vivo, with RNA emerging as the most stable third strand [37, 38]. These DNA-RNA hybrid structures, termed R-loops, have increasingly been identified to regulate DNA replication, transcription, translation, chromatin structure, and genomic stability. Telomeres, composed of nucleoprotein heterochromatin containing 5'-TTAGGG-3' repeats, safeguard eukaryotic chromosome ends from uncontrolled fusions and recombination [38]. Telomerase reverse transcriptase (TERT) elongates telomere ends, while double-stranded T-loop structures formed within telomere regions protect against inappropriate homologous recombination. Additionally, non-coding RNA (TERRA) transcribed from subtelomeric regions hybridizes with telomeric regions, forming R-loop structures that mitigate the risk of homologous recombination-induced conflicts, DNA breaks, and genomic instability. Studies have shown that RNAse H1 deletion increases TERRA accumulation in short telomeres and inhibits cellular senescence. Conversely, R-loop formation and hybridized TERRA levels increase by activating the alternative telomere extension (ALT) pathway in telomerase-negative tumor cells [38]. Under normal physiological conditions, R-loop formation increases during cell division, and eukaryotic RNAse H enzymes degrade accumulated hybridized TERRA molecules, dissolving R-loop structures. Additionally, the RAD51 recombinase protein exhibits high binding affinity to TERRA, mediating its binding to telomeric DNA regions, particularly enhancing R-loop formation in short telomeres.

Hybrid TERRA transcript levels exhibited a notable increase in sperm samples from the chronic mTBI group. This observation aligns with previous findings indicating that telomeres undergo elongation, leading to the accumulation of TERRA, a subtelomeric transcript, particularly in instances of shortened telomeres. Moreover, our observations are consistent with the inverse correlation between TL and TERRA levels observed in somatic cells. [28].

A study by Liu and colleagues in 2019 found that TERRA is transmitted from sperm to zygotes across generations and causes telomere shortening, suggesting that TERRA plays an important role in paternal stress-induced telomere shortening in offspring [39].

In this study, TERRA free fraction levels increased in sperm samples from the acute mTBI group. Additionally, hybrid TERRA levels were found to be increased in sperm samples from the chronic mTBI group. Consistent with the data obtained in this study, we suggest that changes in total and hybrid levels of TERRA regulate TL and that these changes could have effects on the next generation. Transgenerational studies need to be conducted to see what kind of impact it will have.

For TERRA to be fully functional, it must hybridize at telomere ends after transcription. In the event of a DNA double-strand break, the assembly of TERRA at the telomere region and the formation of R-loops depend on the *Rad51* protein (Rad51 recombinase) and the 5′-UUAGGG-3′ repeats. In our study, the transcript level of the *Rad51* gene increased in spermatozoa from the chronic mTBI group in correlation with the levels of hybrid TERRA. In a study by Feretzaki et al. in 2020, it was shown that *Rad51* is directly involved in the formation of the R-loop of TERRA, that Rad51 binds strongly to TERRA and catalyzes the binding of TERRA to telomeric sequences in vitro, i.e. *Rad51*-TERRA is essential for the formation of DNA-RNA hybrid [40]. As a result of our study, *Rad51*, increased in correlation with TERRA in hybrid in the chronic mTBI group.

Due to the triple flux of guanine in the telomere tandem repeat (TTAGGG), telomeric DNA has a high propensity to form these structures, and G-quadruplexes interfere with the replication machinery. Therefore, proper opening and replication of these regions are paramount for telomere integrity and genomic stability. To overcome their unique replication difficulties, telomeres act as a complex of many helicases and nucleases to break down the secondary structures formed at the replication fork and ensure their correct replication [41].

Our findings indicate a significant increase in the transcript levels of RNase H1 in the acute mTBI group and RNase H2 in the spermatozoa of both acute and chronic mTBI groups compared to the control group. This observation aligns with the literature, which emphasizes the crucial role of RNase H enzymes in maintaining genome replication by removing DNA-RNA hybrids, particularly R-loops, which are essential for protecting repetitive DNA regions from DNA double-strand breaks (DSBs).

RNase H1, also known as RNase HI in prokaryotes and RNase H1 in eukaryotes, and RNase H2, known as RNase HII in prokaryotes and RNase H2 in eukaryotes, belong to two major families of RNase H enzymes. While RNase H2 has strict cell cycle regulatory requirements, primarily functioning in G2/M for R-loop processing and ribonucleotide excision repair, RNase H1 can operate independently of the cell cycle to remove R-loops and can be activated in response to high R-loop loads [42, 43]. Recent studies have underscored the involvement of RNase H2 in the excision of single ribonucleotides embedded in genomic DNA and in the removal of R-loops, highlighting its critical role in maintaining genome integrity by facilitating DNA repair and R-loop removal [44].

Given the detrimental effects of non-physiological accumulation of R-loops on genome integrity, including transcriptional and replicative stress leading to DNA double-strand breaks, the upregulation of *RNase H1* and *RNase H2* in response to mTBI suggests a cellular response mechanism to mitigate potential genomic instability. Moreover, the essential requirement of both *RNase H1* and *RNase H2* during mammalian embryogenesis underscores their indispensable roles in early development and further underscores the significance of our findings in the context of reproductive health and potential transgenerational impacts.

Our study findings indicate a significant increase in transcript levels of the *Exo1* gene specifically within sperm samples from the acute and chronic mTBI groups compared to controls. This observation raises intriguing questions about the potential relationship between elevated Exo1 expression and telomerase activity in spermatozoa following TBI. *Exo1*, a gene encoding a protein essential for maintaining telomeres by facilitating RNA removal, plays a pivotal role in averting replication stress induced by linked hybrids between telomeres or other chromosomal regions [41]. Therefore, the observed increase in *Exo1* transcript levels post-mTBI suggests a cellular response mechanism aimed at mitigating potential genomic instability in sperm cells.

The connection between TBI-induced changes in Exo1 expression and telomere maintenance underscores the complex interplay between DNA repair mechanisms and telomere dynamics in the context of TBI-associated reproductive health outcomes. Further investigations are warranted to elucidate the precise mechanisms underlying these observations and their implications for sperm function and offspring health.

Our study findings revealed a significant increase in transcript levels of the tumor suppressor gene Rb1 within sperm samples from the acute r-mTBI, acute mTBI, and chronic TBI groups compared to both the chronic repetitive mTBI groups and the control groups. This observation aligns with previous literature indicating Rb1's crucial role in maintaining telomere integrity and regulating telomere condensation, with Rb1 shown to directly influence the modulation of TERRA transcript levels, thereby impacting telomere architecture [45]. The observed elevation in Rb1 transcript levels post-TBI in our study suggests a potential response mechanism aimed at safeguarding genome integrity in sperm cells following TBI-induced disruption. This finding underscores the complex interplay between Rb1-mediated regulation of telomere integrity and the cellular response to TBI-induced stress. Overall, our results, coupled with existing literature, suggest that the increase in Rb1 transcript levels following TBI may reflect a concerted effort to preserve genome stability and protect against telomere alterations.

The data obtained from our study indicate that TBI exerts effects on sperm transcripts and telomeres. Following a single application of mTBI, we observed significant changes characterized by a pronounced decrease in sperm TL during the acute phase, with diverse responses observed in repetitive applications of mTBI. Interestingly, mTBI induced an increase in TL during the chronic phase of sperm, a phenomenon associated with aging. Furthermore, our findings, in line with existing literature, suggest that TBI leads to alterations in the TERRA free fraction and DNA-RNA hybrid TERRA transcript levels in sperm, indicating impaired telomere function and sperm genome integrity. However, the extent to which TBI influences non-Mendelian inheritance in the embryo and the potential impact of increased TL in sperm on the subsequent generation remain unclear. Considering that TERRA has a telomere protective function, high TERRA levels indicate telomere shortening and serve as a signal for the repair of shortened telomeres [46]. In sperm, except for the chronic mTBI group, the levels are nearly the same as the control group. Particularly, in the r-mTBI acute and chronic groups, which are exposed to prolonged trauma, TERRA may show rapid activity to regulate the current damage situation. At the same time, continuous trauma exposure might create genomic memory in sperm, causing it to have a short-term effect, as seen in the mTBI acute group. According to the data we obtained from the literature and our studies [28, 47, 48], we found that while telomeres shorten in somatic cells, the level of hTERRA in particular increases. However, with this study, we have shown for the first time in the literature how telomere regulation changes in gamete cells after mTBI. Therefore, the role of TERRA in telomere regulation will be better understood with further studies in this field.

In addition to all this, as a result of the correlation analysis, we found that there was a significant negative correlation between hTERRA levels and ACTH, CORT, DHEAS and IGF-1 levels in the mTBI acute group. hTERRA levels may be a biomarker that can be used to monitor sperm damage along with hormone deficiencies caused by mTBI.

In conclusion, investigating the potential transgenerational effects of TBI is essential, necessitating generational tracking in both paternal and maternal lineages using a mouse model. Understanding the non-Mendelian impacts of TBI can provide valuable insights into its pathogenesis, shed light on the mechanisms underlying TBI-induced diseases, and elucidate the etiology of conditions with unknown causes. Given the prevalence of repeated TBI exposure, especially in sports like football and boxing, there is a significant concern for potential paternal transmission of these effects to offspring. Therefore, thorough consideration of the risks associated with repeated TBI exposure, and its potential intergenerational implications is warranted.

## Supplementary Information

Below is the link to the electronic supplementary material.

Supplementary file1 (DOCX 39 kb)

## Data Availability

The data supporting the findings of this study are available on request from the corresponding author.
