# Supplementary material for: Impact of mild traumatic brain injury (mTBI) on sperm genome integrity: insights from a mouse model
Source: J Endocrinol Invest. 2025 Mar 10;48(6):1443–56. doi: 10.1007/s40618-025-02549-w (PMC12226631; doi:10.1007/s40618-025-02549-w)
Supplement: Supplementary file 1 — Supplementary file1 (DOCX 39 kb) [file 40618_2025_2549_MOESM1_ESM.docx]

**Impact of Mild Traumatic Brain Injury (mTBI) on Sperm Genome Integrity: Insights from a Mouse Model**

**Supplementary Materials**

**Supplementary Table** **1**. Sequences of primers used to determine telomere lengths.

| **Oligomers** | | **Species** | **Oligomer sequence (5'→3')** | **Amplicon size** |
| --- | --- | --- | --- | --- |
| **Standards** | Telomere Standard | Mus musculus (*Balb/C*) | (TTAGGG)*_14_* | 84 bp |
|  | *36B4*  Standard | Mus musculus | ACTGGTCTAGGACCCGAGAAGACCTCCTTCTTCCAGGCTTTGGGCATCACCACGAAAATCTCCAGAGGCACCATTGA | 79 bp |
| **PCR Primers** | Telomere F | Mus musculus | CGGTTIGTTTGGGTTTGGGTTTGGGTTTGGGTTTGGGTT | 39 bp |
|  | Telomere R | Mus musculus | GGCTTGCCTTACCCTIACCCTTACCCTTACCCTTACCCT | 39 bp |
|  | 36B4 F | Mus musculus | ACTGGTCTAGGACCCGAGAAG | 21 bp |
|  | 36B4 R | Mus musculus | TCAATGGIGCCICTGGAGATT | 21 bp |

**Supplementary Table 2.** Telomere and 36B4 standard series.

| ***Telomere* Standard Series** | **Amount** | **ddH_2_O (**$\mu l$**)** | **Telomere sequence (kb)** |
| --- | --- | --- | --- |
| **Standard 1 (S1)** | 2 $\mu l$ Telomer (10 pmol) standard | 18 | ${1.18\times10}^{7}$ |
| **Standard 2 (S2)** | 2 $\mu l$ S1 | 18 | ${1.18\times10}^{6}$ |
| **Standard 3 (S3)** | 2 $\mu l$ S2 | 18 | ${1.18\times10}^{5}$ |
| **Standard 4 (S4)** | 2 $\mu l$ S3 | 18 | ${1.18\times10}^{4}$ |
| **Standard 5 (S5)** | 2 $\mu l$ S4 | 18 | ${1.18\times10}^{3}$ |
| **Standard 6 (S6)** | 2 $\mu l$ S5 | 18 | ${1.18\times10}^{2}$ |

| ***36B4*  Standard Series** | **Amount** | **ddH_2_O  (**$\mu l$**)** | **36B4  sequence (kb)** |
| --- | --- | --- | --- |
| **Standard 1 (S1)** | 2 $\mu l$ 36B4 (10 pmol) standard | 18 | ${2.63\times10}^{8}$ |
| **Standard 2 (S2)** | 2 $\mu l$ S1 | 18 | ${2.63\times10}^{7}$ |
| **Standard 3 (S3)** | 2 $\mu l$ S2 | 18 | ${2.63\times10}^{6}$ |
| **Standard 4 (S4)** | 2 $\mu l$ S3 | 18 | ${2.63\times10}^{5}$ |
| **Standard 5 (S5)** | 2 $\mu l$ S4 | 18 | ${2.63\times10}^{4}$ |
| **Standard 6 (S6)** | 2 $\mu l$ S5 | 18 | ${2.63\times10}^{3}$ |

**Supplementary Table 3**. Real Time qPCR conditions for Determination of Telomere Length.

| **Real Time qPCR Program** | | |
| --- | --- | --- |
| 95^o^C | 10 min | 40 Cycle |
| 94 ^o^C | 20 sec |  |
| 60 ^o^C | 20 sec |  |
| 72 ^o^C | 45 sec |  |
| 95 ^o^C | 15 sec | Melting Curve |
| 67 ^o^C | 01 sec |  |
| 40 ^o^C | 30 sec | Cooling |

**Supplementary Table 4.** Primary sequences of genes whose expression level was determined.

| **Gene** | **GenBank Accession** |  | **Oligomer sequence (5'→3')** | **Amplicon size** |
| --- | --- | --- | --- | --- |
| ***Rad51*** | NM_011234 | **F** | GTCCACAGCCTATTTCACGGT | 94 bp |
|  |  | **R** | ACAGCCTCCACTGTATGGTAAC |  |
| ***Exo1*** | NM_012012 | **F** | TTATGGGGTCAAGCCGATTCT | 95 bp |
|  |  | **R** | GGTTGCTTTGTCGTCTCTCTC |  |
| ***Rb1*** | NM_009029 | **F** | TCGATACCAGTACCAAGGTTGA | 95 bp |
|  |  | **R** | ACACGTCCGTTCTAATTTGCTG |  |
| ***RNase H1*** | NM_011275 | **F** | TGGTTCAAAAGGGCAGGAAAG | 163 bp |
|  |  | **R** | AAATGCGTCCTTGCTCACTAC |  |
| ***RNase H2*** | NM_027187 | **F** | TCTACGCCATCTGTTACTGCC | 94 bp |
|  |  | **R** | TCCCGCTCGTTCTCTGTCAA |  |
| ***Gapdh*** | NC_000072.7 | **F** | GTGTTCCTACCCCCAATGTG | 259 bp |
|  |  | **R** | GTCATTGAGAGCAATGCCAG |  |

**Supplementary Table 5:** Demonstration of the relationship between telomere length, TERRA and TERRA expression levels, gene expression and hormone levels in the mTBI acute group by correlation analysis

**Supplementary Table 6:** Demonstration of the relationship between telomere length, TERRA and TERRA expression levels, gene expression and hormone levels in the mTBI chronic group by correlation analysis

**Supplementary Table 7:** Demonstration of the relationship between telomere length, TERRA and TERRA expression levels, gene expression and hormone levels in the r-mTBI acute group by correlation analysis

**Supplementary Table 8:** Demonstration of the relationship between telomere length, TERRA and TERRA expression levels, gene expression and hormone levels in the r-mTBI chronic group by correlation analysis
